# Mu rhythm suppression over sensorimotor regions is associated with greater empathic accuracy

**DOI:** 10.1093/scan/nsac011

**Published:** 2022-02-07

**Authors:** Shir Genzer, Desmond C Ong, Jamil Zaki, Anat Perry

**Affiliations:** Department of Psychology, Hebrew University of Jerusalem, Jerusalem 9190501, Israel; Department of Information Systems and Analytics, National University of Singapore, Singapore 117418, Singapore; Institute of High Performance Computing, Agency for Science, Technology and Research, Singapore 138632, Singapore; Department of Psychology, Stanford University, Stanford, CA 94305, USA; Department of Psychology, Hebrew University of Jerusalem, Jerusalem 9190501, Israel

**Keywords:** empathic accuracy, Mu suppression, inference, EEG, affective cognition

## Abstract

When people encounter others’ emotions, they engage multiple brain systems, including parts of the sensorimotor cortex associated with motor simulation. Simulation-related brain activity is commonly described as a ‘low-level’ component of empathy and social cognition. It remains unclear whether and how sensorimotor simulation contributes to complex empathic judgments. Here, we combine a naturalistic social paradigm with a reliable index of sensorimotor cortex-based simulation: electroencephalography suppression of oscillatory activity in the mu frequency band. We recruited participants to watch naturalistic video clips of people (‘targets’) describing emotional life events. In two experiments, participants viewed these clips (i) with video and sound, (ii) with only video or (iii) with only sound and provided continuous ratings of how they believed the target felt. We operationalized ‘empathic accuracy’ as the correlation between participants’ inferences and targets’ self-report. In Experiment 1 (US sample), across all conditions, right-lateralized mu suppression tracked empathic accuracy. In Experiment 2 (Israeli sample), this replicated only when using individualized frequency-bands and only for the visual stimuli. Our results provide novel evidence that sensorimotor representations—as measured through mu suppression—play a role not only in low-level motor simulation, but also in higher-level inferences about others’ emotions, especially when visual cues are crucial for accuracy.

Imagine that you are consoling a crying friend: her emotional expressions, her tone of voice and what she says all provide rich cues to how she feels, which you naturally piece together to understand her experience. Our inferences about each other are often quite accurate (Zaki and Ochsner, 2011), although imperfect (Eyal *et al.*, 2018).

Empathy is a multifaceted phenomenon (e.g. Decety and Meyer, 2008; Zaki, 2014), and people can draw on multiple empathic processes when evaluating others’ emotional and mental states. One such process is ‘experience sharing’, which refers to people’s tendency to vicariously share the internal states of others (e.g. Levenson and Ruef, 1992). Another is ‘mentalizing’, involves a reasoning component, by which people use their knowledge of the world (their intuitive theories of other people) to reason about others’ emotions, intentions, beliefs and behaviors (Ong *et al.*, 2015; Saxe and Houlihan, 2017). Experience sharing and mentalizing rely on dissociable systems of brain regions, and these processes are triggered preferentially by different classes of social cues (Van Overwalle and Baetens, 2009; Zaki and Ochsner, 2012). Brain regions engaged by mentalizing processes (such as the medial prefrontal cortex, mPFC) are preferentially activated by reading cues describing how others’ emotional and mental states arise in context (e.g. Skerry and Saxe, 2015). By contrast, experience sharing engages brain regions such as the anterior insula and anterior cingulate cortex for pain (e.g. Singer *et al.*, 2004), or parietal and premotor regions for cues about others’ sensorimotor states, such as photographs of facial expressions or motor actions (e.g. Keysers *et al.*, 2010).

A well-established neural signature of experience sharing is mu suppression, measured via electroencephalography (EEG) or magnetoencephalography (for a meta-analysis, see Fox *et al.*, 2016). Neurons in the sensorimotor cortex tend to fire synchronously at rest, resulting in oscillations in the range of 8–13 Hz, often termed mu rhythms. Suppression of these mu rhythms, resulting from increased sensorimotor activity (event-related desynchronization), occurs both when executing motor actions and when observing similar motor actions in others (Pineda, 2005; Perry and Bentin, 2009; Fox *et al.*, 2016). Previous studies have found increased mu suppression when viewing and making judgments about social stimuli, such as perceiving intentionality and emotions from motion (Perry *et al.*, 2010b), viewing emotional facial expressions (Moore *et al.*, 2012; Popov *et al.*, 2013; Rayson *et al.*, 2016; Ensenberg *et al.*, 2017), viewing others’ pain (Perry *et al.*, 2010a), playing a game with others (Perry *et al.*, 2011) and making mental-state attributions (Pineda and Hecht, 2009; Gutsell *et al.*, 2020). It is correlated with trait measures of empathic concern (DiGirolamo *et al.*, 2019) and inversely correlated with dehumanization (Simon and Gutsell, 2021).

Most previous studies examined the relationship between mu suppression and empathy through visual stimuli. However, in real life, when we interact with other people and try to understand them, we often not only see them but also hear them. Furthermore, in some cases, like a phone call, we can only hear the other. Therefore, it is essential to investigate if mu suppression is associated with empathy in general or if this association depends on the information presented in the stimulus. A few studies have investigated the role of mu suppression in processing social auditory stimuli (e.g. Hobson and Bishop, 2017). However, these auditory tasks mainly focus on discriminating speech in noise (Cuellar *et al.*, 2012; Jenson *et al.*, 2014), or listening to language describing actions *vs* abstract concepts (e.g. Moreno *et al.*, 2015). These results do not yet speak to how these processes contribute to the semantic understanding required for more complex empathic inferences. Therefore, in the current manuscript, we want to investigate not only the relationship between mu suppression and empathic inferences but also the influence of the information channels of the stimuli on this association.

The mounting evidence from the literature suggests that mu suppression is linked to inferences about others from low-level visual motor cues such as photographs and possibly low-level auditory cues (see Simon and Gutsell, 2021, for an exception). But the identification and discrimination tasks used in these previous experiments fall short of the complexity of everyday affective reasoning, and it is not clear whether or how these representations contribute to higher-level reasoning about others’ affective states, especially in naturalistic contexts (e.g. Zaki *et al.*, 2008; Ong *et al.*, 2015). Thus, we designed the current study to test the hypothesis that motor representations of others’ actions and expressions, as indexed by mu suppression, support people’s ability to draw ‘accurate’ inferences about others’ affect in naturalistic contexts (Levenson and Ruef, 1992; Zaki *et al.*, 2009b).

In the first experiment (set in the USA), we adapted a task that we had previously used (Zaki *et al.*, 2009a) in which participants (‘observers’) watch videos of other people (‘targets’) recounting emotional autobiographical stories (Figure 1A). As observers are watching these videos, they provide continuous ratings of targets’ affective state throughout the video. Targets’ continuous ratings of their own affect were previously collected, enabling calculation of a measure of empathic accuracy for each observer watching each video (Zaki *et al.*, 2008, 2009a, 2009b). Observers in the current study were shown these autobiographical stories in three viewing conditions: they rated the targets’ affect while watching a muted video (i.e. using only visual information, ‘Video-Only’), while only listening to the sound with no video (i.e. using only auditory information, ‘Audio-Only’) or while watching the video with audio (i.e. with both channels of information, ‘Audio-Video’). Similar to previous studies (Gesn and Ickes, 1999; Hall and Schmid Mast, 2007; Jospe *et al.*, 2020), we hypothesized that observers will perceive another’s affective state better than chance when having just the visual information and significantly better when auditory (linguistic) information is present (Zaki *et al.*, 2009b; Jospe *et al.*, 2020). Going beyond previous studies that looked only at identification and discrimination of simple social stimuli, we were interested in how mu suppression contributes to complex emotional inferences. Thus, insofar as mu suppression tracks processing of emotionally relevant information across both visual and auditory modalities, we hypothesized that mu suppression should contribute to more ‘accurate’ empathic judgments, across all conditions. Moreover, we hypothesized that the correlation between mu suppression and empathic accuracy will be the most substantial in the muted video-only condition. Finally, most prior studies examined mu suppression during short stimuli presentations (e.g. static photographs or 2 s-long video clips; see Fox *et al.*, 2016, for a meta-analysis), so there is almost no evidence on the temporal dynamics of mu and the accuracy of social understanding. Naturalistic emotional understanding in particular fluctuates in small time intervals (Zaki *et al.*, 2008; Devlin *et al.*, 2016). Therefore, we wanted to test the hypothesis that mu suppression during shorter time intervals is related to more accurate affect judgments within that interval.

**Fig. 1.**
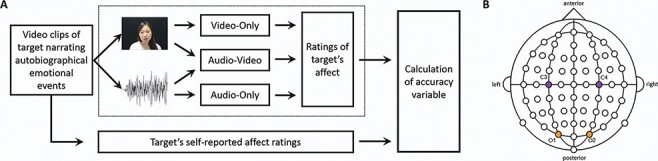
(A) Schematic of the task that participants performed (outlined in the dashed box). Participants are presented with multiple video clips in each of three viewing conditions: Video-Only (with no audio), Audio-Video and Audio-Only (with no video). They provide continuous ratings of how they thought the target in the video felt. Together with the target’s self-reported affect ratings, which we collected previously, we can calculate the accuracy variables that are our dependent measures. (B) Illustration of the four sites that were analyzed: C3 and C4 in the Central region, and O1 and O2 in the Occipital region.

In the second experiment, we aimed to replicate the results of Experiment 1 using a larger sample size and a different stimuli set, in a different language and culture. Therefore, the procedures were identical to Experiment 1, except that Experiment 2 was set in Israel, using Israeli stimuli in Hebrew (Jospe *et al.*, 2020).

## Experiment 1

### Methods

#### Participants

We recruited 21 English-speaking undergraduate students from the University of California, Berkeley, who received course credit for participating in the experiment. We excluded one participant from analysis due to technical problems with the EEG recordings, resulting in a final sample of 20 (18 female, mean age = 20.20 years, s.d. = 2.30; information about handedness was not collected), from diverse ethnic backgrounds (1 American Indian, 8 East Asian, 1 Pacific Islander, 5 White, 5 Latin, 5 Southeast Asian, collected as self-reports from the participants and following the guidelines suggested by Flanagin *et al.*, 2021). All participants reported normal or corrected to normal visual acuity and had no history of psychiatric or neurological disorders as confirmed by a screening interview.

#### Stimuli

We used videos collected as part of a previous project (Ong *et al.*, 2021). Research volunteers (hereafter, ‘targets’; *N* = 68; 40 female, 26 male, 2 not reported; mean age 23.2 years) participated in exchange for monetary compensation and gave their informed consent as approved by the Stanford University Institutional Review Board. Targets were video-recorded narrating three positive and three negative autobiographical emotional events from their lives. After they finished recording these events, targets then watched their own videos and gave a continuous rating of how positive or negative they felt while speaking, using a 100-point rating slider (with endpoints ‘very negative’ to ‘very positive’). The slider allowed targets to continuously update their affect ratings during the video (see Ong *et al.*, 2021, for more details on the stimuli recording and the targets rating procedure; Zaki *et al.*, 2008; Jospe *et al.*, 2020 for a similar approach). We selected nine videos, all containing unique targets from this library. We chose stories that were comprehensible, with at least some facial expressions, and which did not include any names of people, and balanced the number of videos with mostly negative (4), mostly positive (3), and both negative and positive content (2), and the number of videos with male (4) and female (5) targets. For technical reasons, one of these videos did not have the target’s continuous valence rating, so an empathic-accuracy score could not be computed for this video (see below). The length of the videos ranged between 1 min 45 s and 3 min 24 s, with an average of 2 min 22 s. These nine videos were then grouped into three between-subjects sets of equal duration (range: 425–431 s), such that participants in the present study saw a similar duration of audio-only, visual-only or audiovisual stimuli. The assignment of these sets to condition (i.e. which videos were audio-only, visual-only or audiovisual) was counterbalanced across participants.

#### Task

We used a modified version of an empathic-accuracy task that has been used before on several occasions (Devlin *et al.*, 2016; Zaki *et al.*, 2008, 2009a, 2009b, see Figure 1A). In the first half of the session, we recorded EEG from participants while they passively viewed the nine videos. Participants sat approximately 80 cm from the screen and were instructed to carefully notice how the target in the video feels at every moment in time, paying special attention to the momentary changes in the target’s emotion. Each participant saw three video clips in the Audio-Video condition, three clips in the Video-Only condition and three clips in the Audio-Only condition, in a randomized order (see the ‘Stimuli’ section above). All stimuli were preceded by a 5 s fixation point. We interspersed attention checks throughout the task (after every two or three videos), where participants had to answer a question about the video’s content (e.g. ‘In the previous story, what was the mother diagnosed with?’). Note that the attention checks followed only the audio-only or audiovisual videos, as the visual-only videos had no semantic content.

Following this, participants viewed the same videos again, in the same order, without EEG. This time, participants provided continuous ratings of how positive or negative they thought the target felt while speaking, using a 100-point rating slider, from ‘very negative’ to ‘very positive’. This active rating portion was done outside the EEG setup to avoid contamination of the EEG signal—and especially of mu suppression—by the motor movement associated with making ratings. Note that the blocks’ order (i.e. the EEG recording and continuous ratings) was fixed to ensure that the EEG signal would be most spontaneous and not be affected by habituation or prediction. Of course, the behavioral rating may have been affected by these (see the ‘Discussion’ section).

#### EEG data acquisition

We recorded EEG continuously (from DC with a low-pass filter set at 100 Hz) from 64 Ag-AgCl pin-type active electrodes mounted on a Biosemi elastic cap (http://www.biosemi.com/headcap.htm). Recording was done according to the extended 10–20 system. In addition, we recorded from two electrodes placed at the right and left mastoids. During recording, all electrodes were referenced to the common-mode signal electrode between POz and PO3; they were subsequently re-referenced digitally (see EEG data processing). To monitor eye movements and blinks, we measured bipolar horizontal and vertical electrooculography (EOG) derivations using two electrode pairs. One pair was attached to the outer canthi of both eyes, while the other was attached to the infraorbital and supraorbital regions of the right eye. We digitally amplified and sampled at 1024 Hz, both EEG and EOG using a Biosemi Active II system (www.biosemi.com).

#### EEG data processing

We analyzed the EEG data using the Brain Vision Analyzer software (Brain Products). We filtered the raw EEG data using a 0.5 Hz high-pass filter, a 30 Hz low-pass filter (24 dB) and a notch filter at 60 Hz. Following filtering, the data were re-referenced offline to the average signal from the mastoid electrodes. We corrected EEG deflections resulting from eye movements and blinks using Interdependent Component Analysis (Jung *et al.*, 2000), and we removed any remaining artifacts that exceeded plus minus 100 microvolts in amplitude. We segmented each video into 3 s time windows (‘epochs’), as previous mu studies have shown that mu suppression can be reliably estimated in these intervals (e.g. see Fox *et al.*, 2016). We used the first 3 s epoch of each video to serve as a baseline for that video. For each epoch, we used a Fast Fourier Transform (FFT) at 0.5 Hz intervals and with a Hanning window to compute the integrated power in the 8–13 Hz range.

#### EEG measures

For our dependent variable, we defined a suppression index as the natural logarithm (ln) of the ratio of the power during each epoch relative to the power during the fixation period preceding that video (i.e. that video’s baseline; e.g. Perry *et al.*, 2010a). We used the ratio of powers, as opposed to a simple subtraction, to control for the variability in absolute EEG power resulting from individual differences in scalp thickness and electrode impedance. In addition, the ratio data are inherently non-normally distributed due to lower bounding, and so we applied a log transform. Greater mu suppression (i.e. less power compared to baseline) indicates more neuronal activation.

We computed suppression indices at four sites—C3 and O1 on the left hemisphere, and C4 and O2 on the right hemisphere—to compare suppression in the 8–13 Hz range between hemispheres and locations (Figure 1). We chose C3 and C4 as they are classic mu rhythm sites (Pineda, 2005), while the two occipital electrodes were chosen to contrast our predicted mu findings with occipital alpha suppression, a strong and well-known phenomenon attributed to visual-attentional mechanisms (Klimesch, 2012).

#### Behavioral measures

We had two dependent variables of interest. The first, what we term ‘empathic accuracy’ following our earlier work (Zaki *et al.*, 2008, 2009a), is a video-level summary of how accurately the participant judged the target’s affect. Specifically, we operationalized this summary using the correlation of the participant’s judgments with the target’s own self-reported affect. As we intend to examine the correlation between the behavioral ratings and the EEG data, we adjusted the time scale of the behavioral data to match the time scale of the EEG data. Therefore, we segmented each video rating into 3 s intervals. The empathic-accuracy extracted measure is the correlation score between the participant’s and the target’s adjusted ratings.

**Fig. 2.**
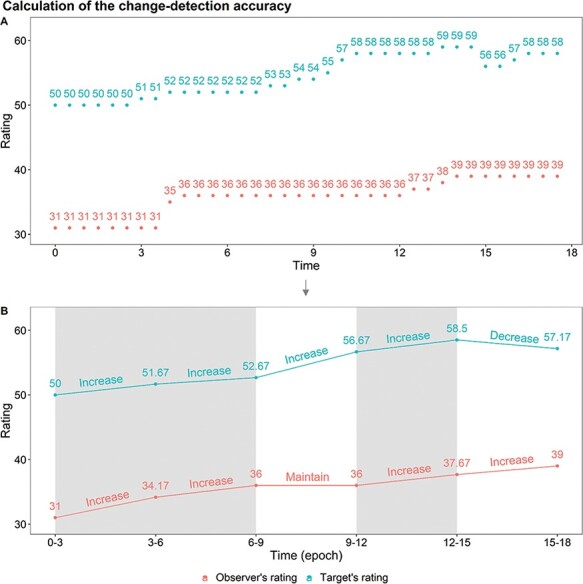
A visualization of the calculation stages of the change-detection accuracy score. (A) A sample of 27 s of target and observer ratings (every 0.5 s). (B) The epoch-level target and observer’s ratings averaged for every 3 s and classification of ratings into one of three categories: an increase in affect, decrease or maintained from the previous epoch. Shaded rectangles indicate epochs where the observer’s rating change (increase, decrease or maintain) matched the target’s rating change.

Our second measure of accuracy, which we term ‘change-detection accuracy’, evaluates how accurately the participant assessed the target’s emotional affect change. To operationalize this measure, we segmented each video rating into 3 s time windows (‘epochs’). For each epoch, we classified participants’ and targets’ ratings into one of three categories: an increase in affect, a decrease or maintained from the previous epoch. We then operationalized change detection such that a ‘successful’ change detection occurred if a participant’s rated change (i.e. increase, decrease or maintain) matched the target’s change at that epoch. If they did not match, this would be a ‘failed’ change detection. Thus, change detection was a binary variable for each epoch that reflected whether the participant successfully detected any change (or lack thereof) in the target’s affect (see Figure 2). Importantly, this definition is ‘scale-invariant’, in that it classifies changes without regard to the magnitude of the change, which helps to mitigate some issues with scale usage as it is not affected by how participants used the scale. Change detection is also ‘memory-less’ such that ratings more than one epoch in the past do not affect this operationalization of accuracy, i.e. as opposed to a correlation, this calculation is not affected by the participant’s accuracy before a given point, as each point is only relative to the one before it.

#### Statistical models

All statistical analyses were performed using R software (R Core Team, 2021). To ensure that the pattern of mu suppression in the central sites differed from those seen in the alpha occipital sites, we used two mixed-effects linear models to predict suppression over the central and the occipital sites with the *lmer* function from the *lme4* package (Bates *et al.*, 2015). For the suppression measures, we averaged the suppression across the whole video, of the central site (averaged across C3 and C4) and the occipital site (averaged across O1 and O2). We added a categorical fixed effect for viewing condition (Audio-Video, Video-Only and Audio-Only). We also added random effects by participant, and by video, to account for the crossed nature of the experiment’s design: each participant saw nine videos, and each video was seen by all participants. To interpret the difference among all the viewing conditions, we conducted *posthoc* contrasts with Bonferroni corrections (Audio-Video *vs* Video-Only, Audio–Video *vs* Audio-Only and Audio-Only *vs* Video-Only) using the *emmeans* function from the *emmeans* package (Lenth *et al.*, 2021).

For the video-level analyses, we used a mixed-effects linear model to predict empathic accuracy using as our main predictor suppression, averaged across the whole video, from C3, C4, O1 and O2. We added a categorical fixed effect for viewing condition (Audio-Video, Video-Only and Audio-Only). We also added random effects by participant, and by video, to account for the crossed nature of the experimental design. To test each variable’s contribution to the model, we used a four-step hierarchical model approach. The first null model had no predictors and had only the random effects by participant and video. The suppression at the electrodes was added to the second model, the viewing condition was added to the third model and the interaction between all the electrodes and the viewing condition was added to the fourth model. Then, we compared the models with F-test estimations based on the Kenward–Roger approach using the function *KRmodcomp* from the *pbkrtest* package (Halekoh and Højsgaard, 2014) to assess each variable’s contribution to the model’s goodness of fit. We interpreted the variable parameters from the most complex model, which significantly improved the model’s goodness of fit, using the *tab_model* function from *sjPlot* package (Lüdecke, 2021), and we conducted *posthoc* contrast comparisons with Bonferroni correction for the viewing-condition contrasts (Audio-Video *vs* Video-Only, Audio-Video *vs* Audio-Only and Audio-Only *vs* Video-Only).

As change detection is a binary variable (success/failure), we used a slightly different approach for the epoch-level analyses. Instead of using a linear mixed-effects model, we used a generalized linear mixed-effects model to predict change detection (i.e. a binomial variable) with the *glmer* function from the *lme4* package (Bates *et al.*, 2015). Then, we used a similar four-step hierarchical model approach with likelihood ratio test comparisons utilizing the *anova* function to assess each variable’s contribution to the model’s goodness of fit. Again, we interpreted the variables’ parameters from the most complex model, which significantly improved the model’s goodness of fit, and we conducted *posthoc* contrast comparisons with Bonferroni correction for the viewing-condition contrasts (Audio-Video *vs* Video-Only, Audio-Video *vs* Audio-Only and Audio-Only *vs* Video-Only).

Note that all analyses were conducted on all data points without outlier removal to maintain as much statistical power as possible due to the small sample size.

#### Data and code availability

All data and code can be found at: https://osf.io/k7bmw/?view_only=08f118913a7946a7ac765fba62391663.

### Results

**Fig. 3.**
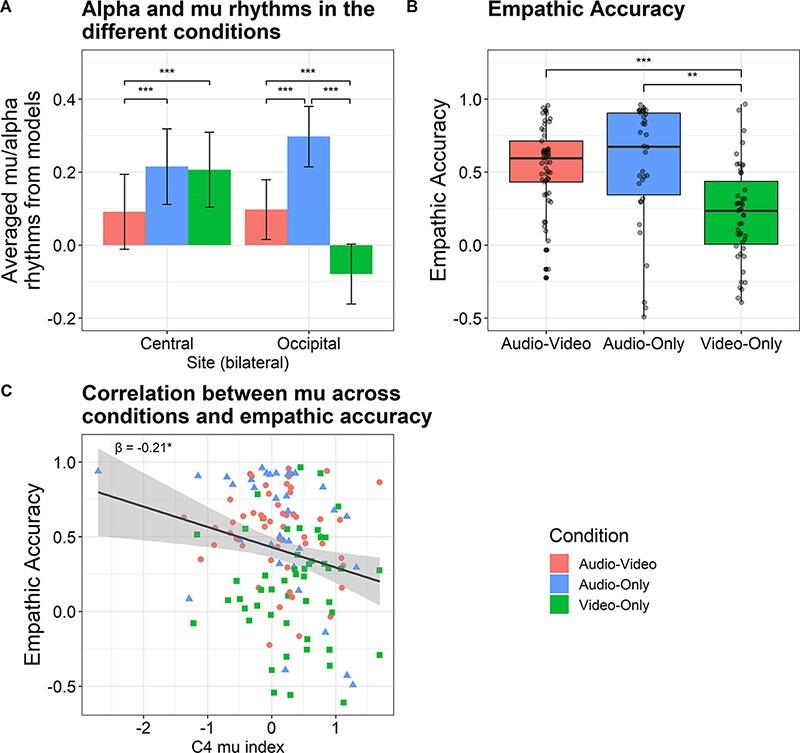
Experiment 1 results. (A) A barplot of mu and alpha rhythms in the different conditions over the central and occipital sites. Values obtained from the linear mixed-effects models predicting participants’ mu and alpha rhythms in the different conditions. More negative values indicate more suppression relative to baseline. The error bars reflect SE. (B) A boxplot of empathic-accuracy scores in the different conditions. Values obtained from the linear mixed-effects models predicting participants’ accuracy at rating the targets’ affect across each video. (C) A scatterplot of video-level empathic accuracy against averaged mu suppression in electrode C4. Data-points are colored by viewing condition. On the horizontal axis, more negative values indicate greater suppression. The line represents a best-fit line, showing a negative correlation between mu suppression and accuracy. There was no interaction of suppression by condition on accuracy. *P < 0.05, **P < 0.01, ***P < 0.001.

First, we examined the levels of suppression across the different sites (Figure 3A). Over the central sites, participants exhibited the greatest mu suppression (less activation) while watching the Audio-Video clips, as compared to the Video-Only clips (β = −0.14, 95% confidence interval [−0.19, −0.09], *t* = −5.77, Bonferroni-corrected *P* < 0.001), and compared to the Audio-Only clips (β = −0.15 [−0.21, −0.10], *t* = −5.40, *P* < 0.001). No difference was found between the Video-Only and Audio-Only conditions (β = −0.01 [−0.07, 0.04], *t* = −0.37, *P* = 1.00). By contrast, over the occipital sites, participants exhibited the greatest alpha suppression (less activation) watching the silent Video-Only clips, as compared to watching the Audio-Video clips (β = −0.20 [−0.25, −0.15], *t* = −7.96, *P* < 0.001), and compared to listening to the Audio-Only clips (β = −0.42 [−0.48, −0.36], *t* = −14.81, *P* < 0.001). There was also greater alpha suppression in the Audio-Video condition compared to the Audio-Only condition, which has no visual information (β = −0.22 [−0.28, −0.17], *t* = −7.85, *P* < 0.001). The different patterns of mu and alpha suppression strengthen the notion that suppression over central sites reflects a different neural phenomenon that is reliant on both the visual and auditory modalities (Pineda, 2005; Le Bel *et al.*, 2009).

Next, we turned to the video-level analyses predicting empathic accuracy. The model comparison indicated that suppression and condition, but not the interaction between them, significantly improved model goodness-of-fit (see Table 1 for model comparisons; for the full model, see Table 2; reporting practices based on Aguinis *et al.*, 2013).

**Table 1.** Model comparison for Experiment 1, assessing the contribution of each variable (suppression at electrodes C3, C4, O1, O2; condition; and the interaction between them) to the goodness-of-fit of the (left) linear mixed-effects model predicting participants’ accuracy at rating the targets’ affect across each video, and the (right) generalized linear mixed-effects model predicting whether the participant’s rating change at the epoch level (increased, decreased or maintained, compared to the previous epoch) matched the target’s rating change

Model comparison		Video-level models: empathic accuracy		Epoch-level models: change detection	
Full model	Restricted model	*F* statistic	*P*-value	${\chi ^2}$ statistic	*P*-value
Suppression model	Null model	${F_{\left( {4,126} \right)}}$ = 4.34	**0.003**	${\chi ^2}{_{\left( 4 \right)}}$ =21.21	**<0.001**
Suppression and condition model	Suppression model	${F_{\left( {2,112} \right)}}$ = 16.51	**<0.001**	${\chi ^2}{_{\left( 2 \right)}}$ = 8.30	**0.02**
Interaction model	Suppression and condition model	${F_{\left( {8,114} \right)}}$ = 0.76	0.64	${\chi ^2}{_{\left( 8 \right)}}$ =11.67	0.17

. σ2 – the random effect variances, τ00 – the random intercept variance, or between-subject variance.

**Table 2.** Summary of statistical models for Experiment 1. Left: results from a linear mixed-effects model predicting participants’ accuracy at rating the targets’ affect across each video. Right: results from a generalized linear mixed-effects model predicting whether participants’ rating change at the epoch level (increased, decreased or maintained, compared to the previous epoch) matched the targets’ rating change

	Video-level models: empathic accuracy				Epoch-level models: change detection			
Predictors	β	SE	CI	*P*	β	SE	CI	*P*
Intercept	0.33	0.21	−0.08, 0.74	<0.001	−0.32	0.10	−0.51, −0.12	0.002
C3 suppression	0.04	0.10	−0.17, 0.24	0.73	−0.03	0.04	−0.11, 0.05	0.47
C4 suppression	−0.21	0.10	−0.40, −0.01	0.04	−0.12	0.04	−0.20, −0.04	0.004
O1 suppression	0.26	0.14	−0.01, 0.53	0.06	0.00	0.05	−0.10, 0.10	0.98
O2 suppression	−0.20	0.14	−0.49, 0.08	0.16	0.09	0.05	−0.02, 0.19	0.10
Video-Only *vs* Audio-Video	−0.86	0.15	−1.16, −0.56	<0.001	−0.17	0.06	−0.30, −0.05	0.005
Audio-Only *vs* Audio-Video	−0.20	0.17	−0.54, 0.13	0.24	−0.03	0.07	−0.17, 0.11	0.65
Random effects								
σ^2^	0.08				3.29			
τ_00_	0.00 _participantID_				0.09 _participantID_			
	0.04 _videoID_				0.03 _videoID_			
ICC	0.35				0.03			
*N*	20 _participantID_				20 _participantID_			
	8 _videoID_				8 _videoID_			
Observations	137				6370			
Marginal *R*^2^/conditional *R*^2^	0.22/0.49				0.01/0.04			
AIC	108.25				8485.02			
Log-likelihood	−44.13				−4233.51			

Notes: See the ‘Methods’ section for operationalization of the dependent variables. β indicates the standardized beta coefficients on suppression at the electrodes, where negative values indicate greater suppression. SE: standard error of the regressor; CI: confidence intervals of the standardized beta coefficients of the regressor; ICC: the intraclass correlation coefficient; AIC: the Akaike information criterion.

When we consider the main effects of condition in the suppression and condition model, there is higher empathic accuracy for the Audio-Video condition than the Video-Only condition (β = 0.86 [0.56, 1.16], *t* = 5.67, Bonferroni-corrected *P* < 0.001). Higher empathic accuracy was also found in the Audio-Only condition compared to the Video-Only condition (β = 0.66 [0.29, 1.02], *t* = −3.56, *P* = 0.002). No difference was found in empathic accuracy between Audio-Video and Audio-Only conditions (β = 0.20 [−0.13, 0.54], *t* = 1.17, *P* = 0.73; Figure 3B; see Jospe *et al.*, 2020, for similar findings). We then considered the main effects of suppression. Greater mu suppression in C4 (i.e. less activation of mu rhythms over the right sensorimotor cortex) was significantly associated with greater empathic accuracy (β = −0.21 [−0.40, −0.01], *t* = −2.04, *P* = 0.04; see Figure 3C). No significant correlation was found between mu suppression in C3 and empathic accuracy or between alpha suppression in O1 and O2 and empathic accuracy (Table 2).

**Fig. 4.**
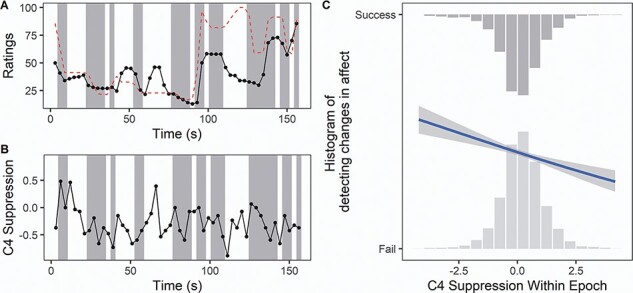
Examining change detection in Experiment 1. (A) Ratings made by a sample participant watching a clip in the Audio-Video condition, compared to the target’s ratings (dashed red line). Ratings range from ‘very negative’ (0) to ‘very positive’ (100). Shaded rectangles indicate epochs where the participants’ rating change (increase, decrease or maintain) matched the target’s rating change. (B) Corresponding suppression index in C4 for the same participant watching the same video clip. More negative values indicate more suppression. (C) This plot, averaged across all participants, shows the histogram of mu suppression in C4 for successful change detections (top histogram) and unsuccessful change detections (bottom histogram). More negative values on the horizontal axis indicate more suppression. The blue line represents a best-fit line from a binomial general linear model, showing a significant negative correlation: More C4 suppression is associated with a greater probability of successfully detecting a change in the target’s affect.

Finally, we considered the epoch-level analyses predicting change detection. Model comparison similarly showed that suppression and condition, but not the interaction between them, significantly improved model goodness-of-fit (see Table 1 for model comparisons; for the full model, see Table 2; reporting practices based on Aguinis *et al.*, 2013; Nakagawa and Schielzeth, 2013). Similar to the video-level model, this model revealed enhanced change detection for the Audio-Video condition compared to the Video-Only condition (β = 0.17 [0.05, 0.30], *t* = 2.79, Bonferroni-corrected *P* = 0.02). However, no significant difference was found between the Audio–Video and Audio-Only conditions (β = 0.03 [−0.11, 0.17], *t* = 0.46, *P* = 1.00), or between the Audio-Only and Video-Only conditions (β = 0.14 [−0.003, 0.29], *t* = −1.92, *P* = 0.16). When we considered the main effects of suppression, consistent with the results for the video-level model, the epoch-level model also indicated that greater mu suppression in C4 (i.e. less activation of mu rhythms over the right sensorimotor cortex) was significantly associated with enhanced change detection (β = −0.12 [−0.20, −0.04], *t* = −2.87, *p* = 0.004; see Figure 4). No significant correlation was found between mu suppression in C3 and change detection, or between alpha suppression in O1 and O2 and change detection (see Table 2).

### Discussion

In Experiment 1, we found that greater mu suppression in electrode C4, over the right sensorimotor cortex, was significantly associated with greater empathic accuracy. This is in line with previous studies that found evidence for right-lateralized mu suppression involving recognition of emotional expressions (Moore *et al.*, 2012; Rayson *et al.*, 2016). Importantly, our results not only provide corroborating evidence that sensorimotor representations—specifically in the right hemisphere—are important in processing emotional information, they also reveal that these sensorimotor representations ‘contribute to accurate affect judgments in complex naturalistic stories’. We did not find an interaction with condition, suggesting that these sensorimotor representations may not be limited to the visual modality and contribute to empathic accuracy across both visual and auditory modalities.

We also found that greater mu suppression over the right sensorimotor cortex (electrode C4) was significantly associated with greater accuracy at detecting ‘epoch-to-epoch’ changes in affect. To the best of our knowledge, this is the first time that mu suppression has been analyzed at such a fine-grained level within a stimulus, revealing sensorimotor sensitivity to changes in complex stimuli on a several-seconds-level resolution. These results, although correlational, strengthen the notion that the sensorimotor cortex may significantly add to understanding complex affective cues as they unfold in a natural environment.

## Experiment 2

Due to the novelty of the previous experiment’s findings, we conducted a second experiment to replicate the results. We used a larger sample size, and a different stimulus set, in a different language and culture, to further generalize the results. Experiment 2 was identical to Experiment 1, but it was conducted in Israel using an Israeli stimuli set, and stories were in Hebrew (Jospe *et al.*, 2020).

### Methods

#### Participants

We recruited 56 Hebrew-speaking undergraduate students from the Hebrew University of Jerusalem, who received either course credit or monetary compensation at a rate of 40 NIS per hour (∼$15) for participating in the experiment. We excluded six participants from analysis due to technical problems during the recordings and four participants due to massive EEG alpha waves, resulting in a final sample of 46 participants (25 female, mean age = 23.68 years, s.d. = 2.05, 43 right-handed). Information about ethnicity was not collected. All participants reported normal or corrected to normal visual acuity and had no history of psychiatric or neurological disorders, as confirmed by a screening interview.

#### Stimuli

We used videos in Hebrew from an Israeli empathic-accuracy stimuli set we collected as part of a previous project, in an identical manner to that described above (for full details, see Jospe *et al.*, 2020). From this set, we selected nine videos, all containing unique targets. We chose stories that were comprehensible, with at least some facial expressions, and which did not include any names of people and balanced the number of male (4) and female (5) targets, with mostly negative (4), mostly positive (3), and both negative and positive content (2). The lengths of the videos were between 2 min 2 seconds and 3 min 48 s, with an average of 2 min 43 s. These nine videos were then grouped into three between-subjects sets of equal duration (range: 454–520 s), such that participants in the present study saw a similar duration of audio-only, visual-only or audiovisual stimuli. The assignment of these sets to condition (i.e. which videos were audio-only, visual-only or audiovisual) was counterbalanced across participants.

#### Task

The empathic-accuracy task was identical to Experiment 1, except for the different stimuli set.

#### EEG data acquisition

Identical to Experiment 1.

#### EEG data processing

We conducted two EEG data-processing procedures: the first was identical to the one in Experiment 1 as a replication. It should be noted that although we generally define mu rhythms as oscillations in the range of 8–13 Hz, the exact numerical boundaries of the mu frequency range are variously defined in the literature as 7–12Hz, 8–13Hz, 9–11Hz (Cohen, 2021) and may be affected by individual differences (Chiang *et al.*, 2011). Therefore, we conducted an exploratory analysis following the initial analysis, in which we extracted individualized 2 Hz frequency bands of mu rhythm for each participant. This method may be more robust for finding effects, instead of a more smeared response that uses the general 8–13 Hz range (see Lepage and Théoret, 2006; Muthukumaraswamy and Johnson, 2004, for similar analyses). Individual mu rhythm bands were defined by the following procedure: for each participant and each video, we averaged the epoched data following the FFT procedure. Then, we averaged this data across videos to compute the average integrated power in the 8–13 Hz range for that participant across conditions. We manually identified the maximum power peak in the 8–13 Hz range and defined the 2 Hz frequency band adequacy (1 Hz above and below the maximum power peak). If the maximum power peak could not be identified, we used 10 Hz as a default and chose 9–11 Hz accordingly. Then, for each participant in each condition, we exported the FFT of that 2 Hz range, at 0.5 Hz intervals and with a Hanning window, to compute the integrated power of the individualized mu rhythm.

#### EEG measures

For our dependent variable, we defined a suppression in four sites (C3, C4, O1, O2) as in Experiment 1, for both the full range and individualized EEG exported data.

#### Behavioral measures

We extracted two dependent variables, the ‘empathic accuracy’ score and the ‘change-detection accuracy’, as in Experiment 1.

#### Statistical models

Similar to Experiment 1 with two adjustments, (i) for the behavioral measures, we removed trials with 2 s.d. away from the overall global mean empathic accuracy (for a similar procedure, see Jospe *et al.*, 2020), which removed 29 data-points out of 402 (7.2%); (ii) following the results of Experiment 1, and in order to increase the statistical power, for the four models’ comparison, the last model with the interaction included interaction between viewing condition and electrode C4 only and not with all electrodes.

### Results

Using the whole 8–13 Hz frequency range did not replicate the findings of Experiment 1 (for the analysis, see supplementary materials)—that is, there was no significant correlation between C4 mu suppression and empathic accuracy across the 8–13 Hz range.

**Fig. 5.**
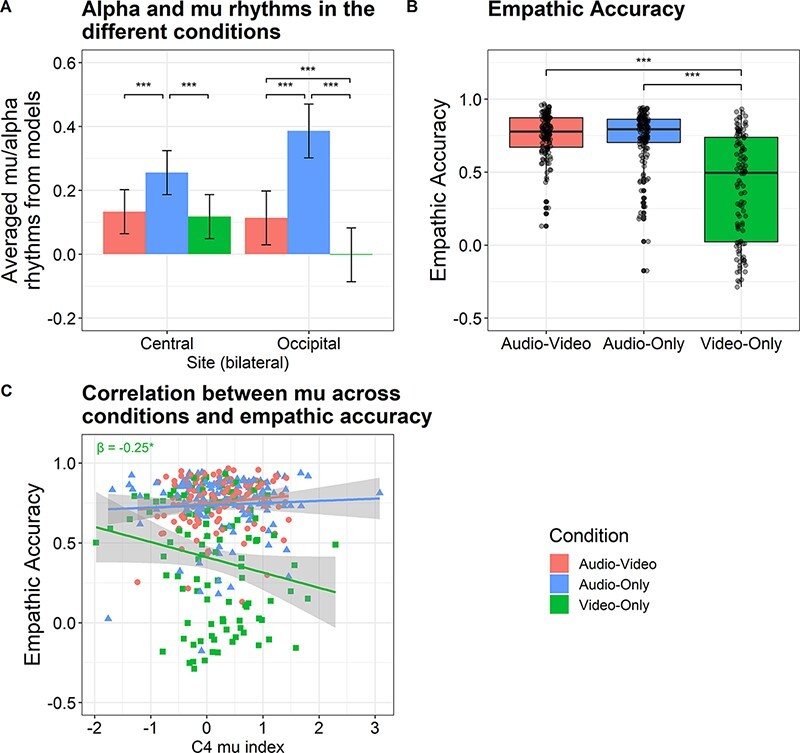
Experiment 2 results. (A) A barplot of mu and alpha rhythms in the different conditions over the central and occipital sites. Values are obtained from the linear mixed-effects models predicting participants’ mu and alpha rhythms in the different conditions. More negative values indicate more suppression, relative to baseline. The error bars reflect SE. (B) A boxplot of empathic accuracy in the different conditions. Values are obtained from the linear mixed-effects model predicting participants’ accuracy at rating a target’s affect across each video. (C) A scatterplot of empathic accuracy by viewing condition against averaged mu suppression in electrode C4. Datapoints are colored by viewing condition. On the horizontal axis, more negative values indicate greater suppression. The line represents a best-fit line, showing a negative correlation between mu suppression and accuracy. *P < 0.05, **P < 0.01, ***P < 0.001.

We next conducted an exploratory analysis using the individual 2 Hz frequency range for each participant (see the ‘Methods’ section), which may be more sensitive due to individual differences in peak frequencies (Muthukumaraswamy and Johnson, 2004; Lepage and Théoret, 2006). Here, similar to Experiment 1, the mixed-effects linear model across the central sites reflected a different suppression pattern compared to the occipital sites (Figure 5A). Over the central sites, we found no difference in mu suppression between the Video-Only and Audio-Video conditions (β = −0.02 [−0.05, 0.01], *t *= −1.29, Bonferroni-corrected *P* = 0.59). However, greater suppression was found for the Video-Only condition compared to the Audio-Only condition (β = −0.17 [−0.20, −0.14], *t* = −11.47, *P* < 0.001). There was also greater mu suppression in the Audio-Video than the Audio-Only condition (β = −0.15 [−0.18, −0.12], *t* = −10.27, *P* < 0.001). By contrast, over the occipital sites, participants exhibited greater alpha suppression when watching the silent Video-Only clips, as compared to when watching the Audio-Video clips (β = −0.12 [−0.15, −0.10], *t* = −8.72, *P* < 0.001) and compared to listening to the Audio-Only clips (β = −0.42 [−0.44, −0.39], *t* = −28.91, *P* < 0.001). There was also greater alpha suppression in the Audio-Video condition compared to the Audio-Only condition (β = −0.29 [−0.32, −0.26], *t* = −20.43, *P* < 0.001).

Next, we turned to the model comparisons predicting empathic accuracy. The model comparisons suggest that suppression, condition and—unlike Experiment 1—the interaction between condition and mu suppression at C4 significantly improved the model goodness-of-fit (see Table 3; for the full model, see Table 4).

**Table 3.** Model comparison for Experiment 2, assessing the contribution of each variable (suppression at electrodes C3, C4, O1, O2; condition; and the interaction between them) to the goodness-of-fit of the (left) linear mixed-effects models predicting participants’ accuracy at rating the target’s affect across each video, and the (right) generalized linear mixed-effects model predicting whether a participant’s rating change at the epoch level (increased, decreased or maintained, compared to the previous epoch) matched the target’s rating change

Model comparison		Video-level models: empathic accuracy		Epoch-level models: change detection	
Full model	Restricted model	*F* statistic	*P*-value	${\chi ^2}$ statistic	*P*-value
Suppression model	Null model	${F_{\left( {4,247} \right)}} $ = 1.55	0.19	${\chi ^2}{_{\left( 4 \right)}}$ =11.24	**0.02**
Suppression and condition model	Suppression model	${F_{\left( {2,330} \right) }}$ =91.62	**<0.001**	${\chi ^2}{_{\left( 2 \right)}}$ =64.47	**<0.001**
Interaction model	Suppression and condition model	${F_{\left( {8,351} \right)}}$ = 3.53	**0.03**	${\chi ^2}{_{\left( 2 \right)}}$ = 0.26	0.88

**Table 4.** Summary of statistical models for Experiment 2. Left: results from a linear mixed-effects model predicting participants’ accuracy at rating the target’s affect across each video. Right: results from a generalized linear mixed-effects model predicting whether a participant’s rating change at the epoch level (increased, decreased or maintained, compared to the previous epoch) matched the target’s rating

	Video-level models: empathic accuracy				Epoch-level models: change detection			
Predictors	β	SE	CI	*P*	β	SE	CI	*P*
Intercept	0.38	0.13	0.12, 0.64	<0.001	−0.29	0.06	−0.41, −0.16	<0.001
C3 suppression	−0.04	0.05	−0.14, 0.07	0.48	0.01	0.02	−0.03, 0.04	0.63
C4 suppression (simple slope in Audio-Video condition)	0.11	0.08	−0.06, 0.27	0.21	−0.01	0.02	−0.04, 0.03	0.70
O1 suppression	−0.01	0.06	−0.12, 0.11	0.89	−0.06	0.02	−0.10, −0.02	0.002
O2 suppression	0.03	0.06	−0.09, 0.16	0.58	0.03	0.02	−0.02, 0.07	0.22
Video-Only *vs* Audio-Video	−1.27	0.10	−1.46, −1.07	<0.001	−0.23	0.04	−0.30, −0.16	<0.001
Audio-Only *vs* Audio-Video	−0.05	0.10	−0.24, 0.14	0.70	0.03	0.04	−0.04, 0.10	0.42
C4 suppression* Video-Only interaction	−0.25	0.11	−0.46, −0.05	0.017				
C4 suppression* Audio-Video interaction	−0.03	0.10	−0.23, 0.16	0.72				
Random effects								
σ^2^	0.05				3.29			
τ_00_	0.00 _participantID_				0.01 _participantID_			
	0.01 _videoID_				0.03 _videoID_			
ICC	0.17				0.01			
*N*	46 _participantID_				46 _participantID_			
	9 _videoID_				9 _videoID_			
Observations	372				20 890			
Marginal *R*^2^/conditional *R*^2^	0.32/0.43				0.005/0.02			
AIC	23.73				28 120.66			
Log-likelihood	0.13				−14 051.33			

Notes: See the ‘Methods’ section for operationalization of the dependent variables. β indicates the standardized beta coefficients on suppression at the electrodes, where negative values indicate greater suppression. SE: standard error of the regressor. CI: confidence intervals of the standardized beta coefficients of the regressor.

* Indicates interaction.

This model revealed again, as in Experiment 1, main effects of condition: higher empathic accuracy for the Audio-Video condition compared to the Video-Only condition (β = 1.27 [1.07, 1.46], *t* = 12.54, Bonferroni-corrected *P* < 0.001) and higher empathic accuracy in the Audio-Only condition compared to the Video-Only condition (β = 1.22 [1.01, 1.42], *t* = −11.72, *P* < 0.001), with no difference between the Audio-Video and Audio-Only conditions (β = 0.05 [−0.14, 0.24], *t *= 0.52, *P* = 1.00; see Figure 5B). However, different from Experiment 1, we found a significant interaction between mu suppression at C4 and condition, such that greater mu suppression at C4 was associated with higher empathic accuracy only for the Video-Only condition compared to the Audio-Video condition (β = −0.25 [−0.46, −0.05], *t* = −2.41, *P* = 0.02; see Figure 5C), and for the Video-Only condition compared to the Audio-Only condition (β = −0.22 [−0.41, −0.03], *t* = −2.31, *P* = 0.02). The simple slope of mu suppression at C4 in the Video-Only condition when controlling for all the other variables was marginally significant (β = −0.15 [−0.30, −0.00], *t* = −1.94, *P* = 0.053). No other variable was significantly correlated with empathic accuracy (Table 4). All results remain the same when including only the 43 right-handed participants (for the analysis, see supplementary materials). In the supplementary, we also added the re-analysis of Experiment 1 with outlier removal. This analysis demonstrates a similar correlation trend between mu suppression and empathic accuracy though not significant. Furthermore, we re-analyzed the results of Experiment 2 without outlier removal. In this analysis, the correlation between mu suppression and empathic accuracy was not found to be significant (for the analysis, see supplementary materials).

Finally, we turned to the epoch-level analysis. Model comparisons suggested that there were no significant interactions of suppression with condition, so in Table 4 we report the model without interactions. When considering the main effects of condition, similar to the video-level model, this model revealed enhanced change detection for the Audio-Video condition compared to the Video-Only condition (β = 0.23 [016, 0.30], *t* = 6.64, Bonferroni-corrected *P* < 0.001). Enhanced change detection was also found in the Audio-Only condition compared to the Video-Only condition (β = 0.26 [0.19, 0.33], *t *= −7.24, *P* < 0.001). No difference was found in change detection between Audio-Video and Audio-Only conditions (β = −0.03 [−0.10, 0.04], *t* = −0.81, *P* = 1.00). This model also indicated that greater alpha suppression in O1 (i.e. less activation of alpha rhythms over the left occipital cortex) was significantly associated with enhanced change detection (β = −0.06 [−0.10, −0.02], *t* = −3.03, *P* = 0.002). No significant correlation was found between alpha suppression in O2 and change detection. Furthermore, no significant correlation was found between mu suppression in C3 and C4 and change detection (Table 4).

Note, that following this analysis, we re-analyzed the data from Experiment 1, extracting individualized 2 Hz frequency bands of mu rhythm for each participant. The results are similar but not identical, and the correlation between mu suppression and empathic accuracy is marginally significant (*P* = 0.119), as well as for the correlation between mu suppression enhanced change detection (*P* = 0.064, for the analysis, see supplementary materials).

Lastly, we investigated whether the change detection score and empathic accuracy scores were correlated and revealed that there is indeed a weak but significant correlation between them in both studies, strengthening the notion that the two measures are related, yet not identical, and enable capturing different aspects of accuracy (see supplementary materials for the full analyses).

### General discussion

Our results are the first to demonstrate that mu suppression tracks accurate emotion judgments in more complex, naturalistic settings, at least when there is only visual information to rely on. This strengthens the role of sensorimotor representations in social cognition, in making social inferences from sensorimotor cues such as facial expressions or body motion (e.g. Perry *et al.*, 2010b; Moore *et al.*, 2012). In Experiment 1, although we found differences in mean levels of mu suppression across the three conditions, we observed that mu suppression was related to empathic accuracy across all conditions, as hypothesized. This suggests that even in the absence of facial expressions (i.e. in the Audio-Only condition), sensorimotor regions were still engaged to produce representations that contribute to accurate empathic judgments. This relates to previous work reporting mirror-neuron sensitivity to auditory cues, both in monkeys and in humans (see Gazzola *et al.*, 2006; Kohler *et al.*, 2002, for evidence from humans and monkeys, respectively). There are several, not mutually exclusive, possible levels of the stimuli that the mu signature may be tracking in the auditory task: first, mu suppression has been shown to be sensitive to concrete action sentences (Moreno *et al.*, 2015) and may be sensitive to actions (including emotional physical reactions) described by the targets. Second, it may be sensitive to low-level paralinguistics cues (e.g. changes in tone or pitch), which could in turn support higher-level empathic inferences. These options could be further investigated in future research.

Importantly though, these results did not fully replicate in Experiment 2. In this experiment, the correlation of empathic accuracy to mu suppression across the three conditions was marginally significant (*P* = 0.053) only when narrowing our focus to the more sensitive individualized mu frequency bands. We additionally found a significant interaction between mu suppression and the Visual-Only condition. There could be several explanations for these discrepancies: Our experiments, as well as those of others (Gesn and Ickes, 1999; Hall and Schmid Mast, 2007; Kraus, 2017; Jospe *et al.*, 2020), demonstrate that empathic-accuracy abilities are primarily dependent on the narrative that comes from the auditory information. It is therefore likely that when the narrative is present, empathic accuracy relies more on mentalizing and thus on other brain regions (such as the Ventromedial prefrontal cortex or the temporoparietal junction; see Atique *et al.*, 2011; Van Overwalle and Baetens, 2009, for reviews). However, in the absence of a narrative, as in the Video-Only condition, the more dominant mechanism may be sensorimotor simulation, which may explain why we found (in Experiment 2) the greater correlation between mu suppression and empathic accuracy in the Video-Only condition.

The second explanation is the difference in the stimuli used. Indeed, in the second experiment, empathic accuracy has much less variance in the auditory conditions, but not in the Video-Only condition. There is less chance of finding any correlation with less variance, which could explain the interaction found between mu suppression and the Video-Only condition. Note that although the average targets’ emotional intensity ratings are mostly similar across the two different experiments (Supplementary Tables S1 and S2), the emotional intensity of the mostly positive clips in experiment 2 is lower than those of Experiment 1, which may contribute to the result differences between the two experiments. The influence of the target emotional valence and intensity, the target and participant gender, as well as other participants’ characteristics (e.g. age, ethnicity and empathy trait) on the correlation between mu suppression and empathic accuracy, should be tested in future studies, either with larger sample sizes or in an experimental design adapted specifically for answering these important questions.

Note that Experiment 1 was conducted on a very diverse US sample with varied ethnic backgrounds and Experiment 2 on an Israeli sample. As the samples’ diversity could potentially moderate the correlation between mu suppression and empathic accuracy, it strengthens the robustness of the findings beyond different races and ethnicity and may even play a role in the discrepancies between the two experiments’ findings. Future studies should investigate if ethnicity, race or other intergroup differences influence the relationship between mu suppression and empathic accuracy. Moreover, as the two samples have a different proportion of females and males, future studies should investigate if gender affects the relationship between mu suppression and empathic accuracy.

The third explanation is the lower power in Experiment 1, which makes it harder to reveal a significant interaction between mu suppression and condition. This suggest that mu suppression may indeed be more strongly related to empathic processes through the visual domain.

The fourth explanation is of course that our results from Experiment 2 represent a more accurate description of the world. Even if this is the case, we now show that in two EEG experiments, using naturalistic stimuli, and across languages and cultures, mu suppression—a proxy for sensorimotor simulation—contributes to accurate empathic judgments. This simulation may be more evident when there is only visual information to rely on. The differences and similarities between the first and second experiments stress the importance of replication and larger samples in EEG studies.

Our findings add to a small but growing set of studies suggesting that both experience sharing and mentalizing systems contribute to making complex, naturalistic judgments. While the current study stresses the role of sensorimotor activation, presumably supporting experience sharing, there is other evidence that both experience sharing and mentalizing are important. In an earlier fMRI study (Zaki *et al.*, 2009b), accurate empathic judgments engaged mentalizing regions like the mPFC, as well as regions thought to support experience sharing such as the premotor cortex. One important difference we note is that in Zaki *et al.*’s (2009b) fMRI study, fMRI and behavioral ratings were collected simultaneously the first and only time the participants saw the videos. In the present study, participants saw all videos twice, the first time with EEG, and the second time for collecting their behavioral rating. This was done in order to avoid contamination of the EEG mu suppression signal, which is highly sensitive to motor movement. This limitation forced us to use this non-ideal experimental design, which had the potential to conceal the role of mu suppression in empathic processes. This, in fact, provides a stronger, more conservative test of the robustness of these sensorimotor representations, as even in this case, EEG mu suppression from the first viewing was correlated with ratings made during the second viewing.

Finally, although we did not predict lateralization, our finding that empathic accuracy is related to mu suppression only over the right sensorimotor cortex corroborates previous studies that found right-lateralized mu suppression in perceiving emotional expressions, both in adults (Moore *et al.*, 2012) and in children (Rayson *et al.*, 2016). This speaks to a larger consensus in the literature (see Adolphs, 2002, for review) that finds right-hemisphere functionalization of emotion recognition from facial expressions (e.g. Killgore and Yurgelun-Todd, 2007) as well as prosody (e.g. Adolphs *et al.*, 2002).

This study has some limitations. First, our second experiment, albeit having a larger sample size, had less variability in some of the behavioral measures and replicated only some of the initial findings from Experiment 1. Second, as mentioned above, to minimize contamination of the EEG data by motor movement, participants saw all videos twice, and the behavioral ratings were recorded only during the second time they saw the video (without EEG), which may have led to biases in their ratings.

To conclude, the current study reveals an EEG measure of sensorimotor representations, indexed by mu rhythm suppression, that contributes to the accuracy of complex naturalistic empathic judgments.

## Supplementary Material

nsac011_Supp
