# Supplementary material for: Mu rhythm suppression over sensorimotor regions is associated with greater empathic accuracy
Source: Soc Cogn Affect Neurosci. 2022 Feb 7;17(9):788–801. doi: 10.1093/scan/nsac011 (PMC9433844; doi:10.1093/scan/nsac011)
Supplement: nsac011_Supp [file nsac011_supp.zip › suppl.docx]

**Supplementary**

|  | **Target gender** | **Target race/ethnic background** | **Target’s mean rating** | **Participants’ mean rating** |
| --- | --- | --- | --- | --- |
| **Mostly negative** | Male | White & Latinx | 27.25 (9.83) | 23.38 (8.34) |
|  | Male | White | 36.45 (7.04) | 37.23 (11.93) |
|  | Male | White | 40.16 (9.13) | 42.05 (10.20) |
|  | Female | White & Latinx | 42.91 (7.41) | 31.33 (14.50) |
| **Both negative and positive content** | Female | White & Latinx | 53.18 (29.35) | 42.14 (12.10) |
|  | Female | Pacific Islander & White & Latinx | 53.91 (7.14) | 40.82 (8.50) |
| **Mostly positive** | Male | White | 81.52 (14.37) | 66.58 (9.56) |
|  | Female | White | 90.25 (8.43) | 68.52 (16.19) |

*Supplementary Table 1.* General information about the stimuli in Experiment 1.

|  | **Target gender** | **Target**  **race/ethnic background** | **Target’s**  **mean rating** | **Participants’ mean rating** |
| --- | --- | --- | --- | --- |
| **Mostly negative** | Male | Israel, Iran | 20.00 (11.14) | 35.98 (12.42) |
|  | Male | Uzbekistan & Europe | 34.69 (33.07) | 37.87 (8.88) |
|  | Female | Iraq & Poland & Romania | 38.72 (6.53) | 32.29 (9.14) |
|  | Female | Europe | 41.59 (12.93) | 40.45 (14.91) |
| **Both negative and positive content** | Female | USA | 50.22 (10.13) | 49.32 (8.93) |
|  | Female | Iraq & Iran | 56.29 (13.46) | 54.66 (12.91) |
| **Mostly positive** | Female | Hungary & Germany | 65.95 (20.40) | 60.69 (9.23) |
|  | Male | No information available | 69.01 (16.60) | 53.33 (11.55) |
|  | Male | Europe | 88.96 (13.96) | 69.49 (8.21) |

*Supplementary Table 2.* Information on the stimuli in Experiment 2.
Note: Like the USA, Israel is a country of former immigrants, therefore, the target race/ethnic background column refers to the target’s family’s countries of origin.

*The correlation between empathic accuracy and change-detection measures:*

To assess the relationship between the empathic accuracy and change-detection measures, we extracted a *mean change-detection score* that assesses how accurately the participant judged the target’s affect change at a video level. We operationalized this as the relative percentage of “successful” change detection for each video.

To investigate the correlation between empathic accuracy and change-detection measures while controlling for the subjects and the videos variance, we used a mixed-effects linear model with the mean change-detection score as a fixed effect and participant and video as random effects.

In Experiment 1, the mixed-effects linear model revealed a significant correlation between empathic accuracy and change-detection, thus a higher mean change-detection score was significantly associated with greater empathic accuracy (*β* = 0.18 95% Confidence Interval [0.03, 0.33], *t* = 2.35, *p* = 0.02). This result was replicated in Experiment 2 (*β* = 0.28 [0.18, 0.37], *t* = 5.52, *p* < 0.001).

These results demonstrate that although the two measures are related, they are not identical and enable us to capture different aspects of accuracy.

*Experiment 2:* *The results for extracted mu frequency using the whole 8-13 Hz range*

We examined the levels of suppression across the different sites. Over the central sites, participants exhibited the greatest mu suppression (less activation) while watching the Video-Only clips, as compared to the Audio-Video clips (β = -0.05, 95% Confidence Interval [-0.08 , -0.02], *t* = -3.18, *p* = 0.004), and as compared to the Audio-Only clips (β = -0.16, [-0.19 , -0.13], *t* = -10.87, *p* <0.001). There was also greater mu suppression in the Audio-Video than the Audio-Only conditions (β = -0.12, [-0.14 , -0.09], *t* = -7.77, *p* <0.001). Over the occipital sites, participants exhibited the greatest alpha suppression (less activation) watching the silent Video-Only clips, as compared to when watching the Audio-Video clips (β = -0.12, [-0.15 , -0.09], *t* = -8.64, *p* <0.001), and as compared to listening to the Audio-Only clips (β = -0.41, [-0.44 , -0.38], *t* = -28.89, *p* <0.001). There was also greater alpha suppression in the Audio-Video condition than the Audio-Only condition, which has no visual information (β = -0.29, [-0.32 , -0.26], *t* = -20.46, *p* <0.001). As mu and alpha demonstrated similar patterns, we could not be certain that suppression over central sites reflects a different neural phenomenon from occipital alpha suppression attributed to visual-attentional mechanisms (Klimesch, 2012).

Next, we turned to the video-level analyses, predicting empathic accuracy. The model comparison indicated that suppression, condition, but not the interaction between the suppression in C4 and condition, significantly improved model goodness of (see Supplementary Table 3).

| Model Comparison | | **Video-Level Models: Empathic Accuracy** | |
| --- | --- | --- | --- |
| Full model | Restricted model | *F statistic* | *p value* |
| **Suppression model** | **Null model** | $F_{(4,247)}$=1.36 | 0.25 |
| **Suppression and condition model** | **Suppression model** | $F_{(2,329)}$=94.07 | **<0.001** |
| **Interaction model** | **Suppression and condition model** | $F_{(2,356)}$=0.85 | 0.43 |

Supplementary Table 3. Model comparison assessing the contribution of each variable (suppression at electrodes C3, C4, O1, O2; condition; and the interaction between them) to the goodness-of-fit of the (Left) linear mixed-effects models predicting participants’ accuracy at rating the target’s affect across each video

Behaviorally, when we consider the main effects of condition in the suppression and condition model, there was higher empathic accuracy for the Audio-Video condition than the Video-Only condition (β = 1.27, [1.07 , 1.46], *t* = 12.47, *p* <0.001). Higher empathic accuracy was also found in the Audio-Only condition compared to the Video-Only condition (β = 1.22, [1.01 , 1.42], *t* = -11.71, *p* <0.001). No difference was found in empathic accuracy between Audio-Video and Audio- Only conditions (β = 0.05, [-0.14 , 0.24], *t* = 0.52, *p* = 1.00). No significant correlation was found between suppression in any of the electrodes' site to empathic accuracy (see Supplementary Table 4). Hence, we decided to extract individualized 2-Hz frequency bands of mu rhythm for each participant (see main text).

|  | **Video-Level Models:**  **Empathic Accuracy** | | | |
| --- | --- | --- | --- | --- |
| *Predictors* | *β* | *SE* | *CI* | *p* |
| Intercept | 0.38 | 0.14 | 0.11 , 0.64 | **<0.001** |
| C3 suppression | 0.02 | 0.06 | -0.08 , 0.13 | 0.66 |
| C4suppression | -0.03 | 0.05 | -0.14 , 0.08 | 0.56 |
| O1 suppression | 0.08 | 0.06 | -0.05 , 0.21 | 0.22 |
| O2 suppression | -0.03 | 0.07 | -0.16 , 0.10 | 0.67 |
| Video-Only vs. Audio-Video | -1.27 | 0.10 | -1.46 , -1.07 | **<0.001** |
| Audio-Only vs. Audio-Video | -0.05 | 0.10 | -0.24 , 0.14 | 0.60 |
| **Random Effects** |  |  |  |  |
| σ^2^ | 0.05 | | | |
| τ_00_ | 0.00 _participantID_ | | | |
|  | 0.01 _videoID_ | | | |
| N | 46 _participantID_ | | | |
|  | 9 _videoID_ | | | |
| Observations | 373 | | | |
| Marginal R^2^ / Conditional R^2^ | 0.35 / NA | | | |
| AIC | 12.68 | | | |
| Log-Likelihood | 3.66 | | | |

Supplementary Table 4. Summary of statistical models. Results from a linear mixed-effects model predicting participants’ accuracy at rating the target’s affect across each video. See Methods for operationalization of the dependent variables. β indicates the standardized beta coefficients on suppression at the electrodes, where negative values indicate greater suppression.

Note: SE: standard error of the regressor. CI: confidence intervals of the standardized beta coefficients of the regressor.

*Re-analysis of Experiment 2 data including only right-handed participants:*

Similarly to the analysis with all the participants, when including only the right handers the model comparisons suggest that suppression, condition, and the interaction between condition and mu suppression at C4 significantly improved the model goodness-of-fit (see Supplementary Table 5).

| Model Comparison | | **Video-Level Models: Empathic Accuracy** | |
| --- | --- | --- | --- |
| Full model | Restricted model | *F* statistic | *p*-value |
| **Suppression model** | **Null model** | $F_{(4,224)}$=1.53 | **0.19** |
| **Suppression and condition model** | **Suppression model** | $F_{(2,300)}$=81.17 | **<0.001** |
| **Interaction model** | **Suppression and condition model** | $F_{(2,318)}$=3 | **0.05** |

Supplementary Table 5. Model comparison for Experiment 2 (including only right handers), assessing the contribution of each variable (suppression at electrodes C3, C4, O1, O2; condition; and the interaction between them) to the goodness-of-fit of the linear mixed-effects model predicting participants’ accuracy at rating the targets’ affect across each video

This model revealed again, a main effects of condition: higher empathic accuracy for the Audio-Video condition compared to the Video-Only condition (*β* = 1.27 [1.06 , 1.48], *t* = 10.79, Bonferroni-corrected *p* < 0.001), and higher empathic accuracy in the Audio-Only condition compared to the Video-Only condition (*β* = 1.21 [0.99 , 1.43], *t* = 9.89, *p* < 0.001), with no difference between the Audio-Video and Audio-Only conditions (*β* = 0.06 [-0.13 , 0.26], *t* = 0.63, *p* = 1.00). We found a significant interaction between mu suppression at C4 and condition, such that greater mu suppression at C4 was associated with higher empathic accuracy only for the Video-Only condition compared to the Audio-Video condition (*β* = -0.24 [-0.45 ,-0.02], *t* = -2.17, *p* = 0.03), and for the Video-Only condition compared to the Audio-Only condition (*β* = -0.22 [-0.42 ,-0.02], *t* = -2.17, *p* = 0.03). The simple slope of mu suppression at C4 in the Video-Only condition when controlling for all the other variables was marginally significant (*β* = -0.13 [-0.29 ,-0.02], *t* = -1.65, *p* = 0.09).

*Re-analysis of experiment 1 data with* *outliers' removal:*

Similar to the analysis of experiment 2, we conducted outliers' removal procedure and removed trials with two standard deviations away from the overall global mean empathic accuracy. This resulted in the removal of 4.38% of the trials.

The model comparisons suggest that suppression, condition, but not the interaction between condition and mu suppression, significantly improved the model goodness-of-fit (see Supplementary Table 6).

| Model Comparison | | **Video-Level Models: Empathic Accuracy** | |
| --- | --- | --- | --- |
| Full model | Restricted model | *F statistic* | *p value* |
| **Suppression model** | **Null model** | $F_{(4,119)}$=3.57 | **0.0087** |
| **Suppression and condition model** | **Suppression model** | $F_{(2,107)}$=17.92 | **<0.001** |
| **Interaction model** | **Suppression and condition model** | $F_{(8,106)}$=1.12 | 0.36 |

Supplementary Table 6. Model comparison assessing the contribution of each variable (suppression at electrodes C3, C4, O1, O2; condition; and the interaction between them) to the goodness-of-fit of the linear mixed-effects models predicting participants’ accuracy at rating the target’s affect across each video.

This model revealed higher empathic accuracy for the Audio-Video condition than the Video-Only condition ($\beta$ = 0.87 [0.57 , 1.17], *t* = 5.68, *p* <0.001). Higher empathic accuracy was also found in the Audio-Only condition compared to the Video-Only condition ($\beta$ = 0.87 [0.49 , 1.25], *t* = 4.49, *p* <0.001). No difference was found in empathic accuracy between Audio-Video and Audio-Only conditions ($\beta$ = 0.001 [-0.35 , 0.35], *t* = 0.004, *p* = 1.00). In this model, the interaction between mu suppression in C4 and empathic accuracy showed a similar trend to the original analysis (without the outliers' removal), though not significant ($\beta$ = -0.15 [-0.35 - 0.05], *t* = -1.48, *p* = 0.142; see Supplementary Table 7).

|  | **Video-Level Models:**  **Empathic Accuracy** | | | |
| --- | --- | --- | --- | --- |
| *Predictors* | *β* | *SE* | *CI* | *p* |
| Intercept | 0.25 | 0.21 | -0.16 , 0.67 | **<0.001** |
| C3 suppression | 0.07 | 0.10 | -0.13 , 0.27 | 0.495 |
| C4 suppression | -0.15 | 0.10 | -0.35 , 0.05 | 0.139 |
| O1 suppression | 0.24 | 0.14 | -0.04 , 0.51 | 0.095 |
| O2 suppression | -0.22 | 0.15 | -0.51 , 0.07 | 0.137 |
| Video-Only vs. Audio-Video | -0.87 | 0.15 | -1.17 , -0.57 | **<0.001** |
| Audio-Only vs. Audio-Video | 0.00 | 0.18 | -0.34 , 0.34 | 0.997 |
| **Random Effects** |  |  |  |  |
| σ^2^ | 0.06 | | | |
| τ_00_ | 0.00 _participant ID_ | | | |
|  | 0.03 _video ID_ | | | |
| N | 20 _participant ID_ | | | |
|  | 8 _video ID_ | | | |
| Observations | 130 | | | |
| Marginal R^2^ / Conditional R^2^ | 0.22 / 0.51 | | | |
| AIC | 66.06 | | | |
| Log-Likelihood | -23.03 | | | |

*Supplementary Table 7.* Summary of statistical models. Results from a linear mixed-effects model predicting participants’ accuracy at rating the target’s affect across each video. See Methods for operationalization of the dependent variables. β indicates the standardized beta coefficients on suppression at the electrodes, where negative values indicate greater suppression.

Note: SE: standard error of the regressor. CI: confidence intervals of the standardized beta coefficients of the regressor.

*Re-analysis of experiment 2 data with the individual 2Hz frequency range without outliers' removal:*

*When analyzing the results of experiment 2 without any outlier removal, the model comparisons suggest that suppression, condition, but not the interaction between condition and mu suppression, significantly improved the model goodness-of-fit (see Supplementary Table 8). Note that this differs from the analysis after outlier removal.*

| Model Comparison | | **Video-Level Models: Empathic Accuracy** | |
| --- | --- | --- | --- |
| Full model | Restricted model | *F statistic* | *p value* |
| **Suppression model** | **Null model** | $F_{(4,279)}$=5.25 | **<0.001** |
| **Suppression and condition model** | **Suppression model** | $F_{(2,353)}$=124.05 | **<0.001** |
| **Interaction model** | **Suppression and condition model** | $F_{(2,374)}$=0.25 | 0.78 |

Supplementary Table 8. Model comparison assessing the contribution of each variable (suppression at electrodes C3, C4, O1, O2; condition; and the interaction between them) to the goodness-of-fit of the linear mixed-effects models predicting participants’ accuracy at rating the target’s affect across each video

This model revealed higher empathic accuracy for the Audio-Video condition than the Video-Only condition ($\beta$ = 1.30 [1.12 , 1.48], *t* = 14.29, *p* <0.001). Higher empathic accuracy was also found in the Audio-Only condition compared to the Video-Only condition ($\beta$ = 1.23, [1.04 , 1.41], *t* = -12.98, *p* <0.001). No difference was found in empathic accuracy between Audio-Video and Audio- Only conditions ($\beta$ = 0.07 [-0.11 , 0.26], *t* = 0.81, *p* = 1.00). The interaction between mu suppression in C4 and empathic accuracy was not significant ($\beta$ = 0.04 [-0.06 , 0.13], *t* = 0.74, *p* = 0.459; see Supplementary Table 9).

|  | **Video-Level Models:**  **Empathic Accuracy** | | | |
| --- | --- | --- | --- | --- |
| *Predictors* | *β* | *SE* | *CI* | *p* |
| Intercept | 0.46 | 0.12 | 0.23 , 0.69 | **<0.001** |
| C3 suppression | -0.05 | 0.05 | -0.15 , 0.04 | 0.294 |
| C4 suppression | 0.04 | 0.05 | -0.06 , 0.13 | 0.459 |
| O1 suppression | -0.05 | 0.05 | -0.16 , 0.06 | 0.346 |
| O2 suppression | 0.11 | 0.06 | -0.01 , 0.22 | 0.062 |
| Video-Only vs. Audio-Video | -1.30 | 0.09 | -1.48 , -1.12 | **<0.001** |
| Audio-Only vs. Audio-Video | -0.07 | 0.09 | -0.25 , 0.11 | 0.420 |
| **Random Effects** |  |  |  |  |
| σ^2^ | 0.10 | | | |
| τ_00_ | 0.00 _participant ID_ | | | |
|  | 0.02 _video ID_ | | | |
| ICC | 0.16 | | | |
| N | 46 _participant ID_ | | | |
|  | 9 _video ID_ | | | |
| Observations | 401 | | | |
| Marginal R^2^ / Conditional R^2^ | 0.38 / 0.47 | | | |
| AIC | 284.01 | | | |
| Log-Likelihood | -132.00 | | | |

*Supplementary Table 9.* Summary of statistical models. Results from a linear mixed-effects model predicting participants’ accuracy at rating the target’s affect across each video. See Methods for operationalization of the dependent variables. β indicates the standardized beta coefficients on suppression at the electrodes, where negative values indicate greater suppression.

Note: SE: standard error of the regressor. CI: confidence intervals of the standardized beta coefficients of the regressor.

*Re-analysis of Experiment 1 data using individualized 2Hz frequency bands approach:*

Similar to the original analysis of Experiment 1 data, the model comparison indicated that suppression and condition, but not the interaction between them, significantly improved model goodness-of-fit (see Supplementary Table 10 for model comparisons; for the full model see Supplementary Table 11).

| Model Comparison | | **Video-Level Models: Empathic Accuracy** | | **Epoch-Level Models: Change Detection** | |
| --- | --- | --- | --- | --- | --- |
| Full model | Restricted model | *F* statistic | *p*-value | $\chi^{2}$ statistic | *p*-value |
| **Suppression model** | **Null model** | $F_{(4,125)}$= 2.55 | **0.04** | $\chi^{2}{}_{(4)}$ =16.85 | **0.002** |
| **Suppression and condition model** | **Suppression model** | $F_{(2,113)}$= 17.08 | **<0.001** | $\chi^{2}{}_{(2)}$ = 9.31 | **0.009** |
| **Interaction model** | **Suppression and condition model** | $F_{(8,114)}$= 0.39 | 0.92 | $\chi^{2}{}_{(8)}$ =10.89 | 0.21 |

Supplementary Table 10. Model comparison for Experiment 1 (including mu suppression data which extracted by individualized 2Hz frequency bands approach), assessing the contribution of each variable (suppression at electrodes C3, C4, O1, O2; condition; and the interaction between them) to the goodness-of-fit of the (Left) linear mixed-effects model predicting participants’ accuracy at rating the targets’ affect across each video, and the (Right) generalized linear mixed-effects model predicting whether the participant’s rating change at the epoch level (increased, decreased, or maintained, compared to the previous epoch) matched the target’s rating change.

Similarly to the original analysis of Experiment 1 data, there is higher empathic accuracy for the Audio-Video condition than the Video-Only condition (*β* = 0.87 [0.58, 1.17], *t* = 5.64, Bonferroni-corrected *p* < 0.001). Higher empathic accuracy was also found in the Audio-Only condition compared to the Video-Only condition (*β* = 0.75 [0.39, 1.11], *t* = -4.06, *p* < 0.001). No difference was found in empathic accuracy between Audio-Video and Audio-Only conditions (*β* = 0.12 [-0.21, 0.45], *t* = 0.72, *p* = 1.00). However, slightly different from the original analysis of Experiment 1 data, the correlation between mu suppression and empathic accuracy is only marginally significant (*β* = -0.14, [-0.32 , 0.04], *t* = -1.56, *p* = 0.119, see Supplementary Table 11).

We then considered the epoch-level analyses predicting change detection. Model comparison similarly showed that suppression and condition, but not the interaction between them, significantly improved model goodness-of-fit (see Supplementary Table 6 for model comparisons; for the full model see Supplementary Table 11).

Similar to the video-level model, this model revealed enhanced change detection for the Audio-Video condition compared to the Video-Only condition (*β* = 0.18 [0.06, 0.31], *t* = 2.94, Bonferroni-corrected *p* = 0.01). However, no significant difference was found between the Audio-Video and Audio-Only conditions (*β* = 0.03 [-0.11, 0.17], *t* = 0.46, p = 1.00), or between the Audio-Only and Video-Only conditions (*β* = 0.15 [0.01, 0.29], *t* = -2.09, *p* = 0.11). However, slightly different from the original analysis of Experiment 1 data, the correlation between mu suppression and enhanced change detection is marginally significant (*β* = -0.07, [-0.15, 0.00], *t* = -1.85, *p* = 0.064, see Supplementary Table 11).

|  | **Video-Level Models:**  **Empathic Accuracy** | | | | **Epoch-Level Models:**  **Change Detection** | | | |  |
| --- | --- | --- | --- | --- | --- | --- | --- | --- | --- |
| *Predictors* | *β* | *SE* | *CI* | *p* | *β* | *SE* | *CI* | *p* |  |
| Intercept | 0.32 | 0.20 | -0.07, 0.70 | **<0.001** | -0.31 | 0.10 | -0.51, -0.11 | **0.002** | |
| C3 suppression | 0.10 | 0.09 | -0.09, 0.28 | 0.293 | -0.06 | 0.04 | -0.14, 0.01 | 0.106 | |
| C4 suppression | -0.14 | 0.09 | -0.32, 0.04 | 0.119 | -0.07 | 0.04 | -0.15, 0.00 | 0.064 | |
| O1 suppression | 0.10 | 0.12 | -0.13, 0.32 | 0.400 | 0.04 | 0.05 | -0.05, 0.13 | 0.394 | |
| O2 suppression | -0.07 | 0.12 | -0.31, 0.16 | 0.544 | 0.05 | 0.05 | -0.05, 0.14 | 0.335 | |
| Video-Only vs.  Audio-Video | -0.87 | 0.15 | -1.17, -0.58 | **<0.001** | -0.18 | 0.06 | -0.31, -0.06 | **0.003** | |
| Audio-Only vs.  Audio-Video | -0.12 | 0.17 | -0.45, 0.21 | 0.467 | -0.03 | 0.07 | -0.17, 0.11 | 0.647 | |
| **Random Effects** |  |  |  |  |  |  |  |  | |
| σ^2^ | 0.08 | | | | 3.29 | | | |  |
| τ_00_ | 0.00 participantID | | | | 0.10 participantID | | | |  |
|  | 0.03 videoID | | | | 0.03 videoID | | | |  |
| ICC | 0.31 | | | | 0.04 | | | |  |
| N | 20 participantID | | | | 20 participantID | | | |  |
|  | 8 videoID | | | | 8 videoID | | | |  |
| Observations | 137 | | | | 6377 | | | |  |
| Marginal R^2^ / Conditional R^2^ | 0.21 / 0.46 | | | | 0.01 / 0.04 | | | |  |
| AIC | 87.30 | | | | 8497.38 | | | |  |
| Log-Likelihood | -33.65 | | | | -4239.69 | | | |  |

Supplementary Table 11. Summary of statistical models including mu suppression data which extracted by individualized 2Hz frequency bands approach for Experiment 1. Left: Results from a linear mixed-effects model predicting participants’ accuracy at rating the targets’ affect across each video. Right: Results from a generalized linear mixed-effects model predicting whether participants’ rating change at the epoch level (increased, decreased, or maintained, compared to the previous epoch) matched the targets’ rating change. See Methods for operationalization of the dependent variables. β indicates the standardized beta coefficients on suppression at the electrodes, where negative values indicate greater suppression. Note: SE: standard error of the regressor. CI: confidence intervals of the standardized beta coefficients of the regressor.
